# Effects of Insect-Resistant Maize HGK60 on Community Diversity of Bacteria and Fungi in Rhizosphere Soil

**DOI:** 10.3390/plants11212824

**Published:** 2022-10-24

**Authors:** Yinxiao Wang, Mingjun Zhang, Shengyan Li, Pengcheng Li, Zhihong Lang

**Affiliations:** Biotechnology Research Institute, Chinese Academy of Agricultural Sciences, Beijing 100081, China

**Keywords:** *cry1ah* transgenic maize, bacterial community composition, fungal community composition, risk assessment, illumina novaseq PE250 sequencing

## Abstract

The influence of biotech crops on microbial communities in rhizosphere soil is an important issue in biosafety assessments. The transgenic maize HGK60 harboring the Bt *cry1Ah* gene enhanced the resistance to lepidopteran pests, while the ecological risk of HGK60 maize on rhizosphere microorganisms is unclear. In this study, we comprehensively analyzed the diversity and composition of bacterial and fungal communities in the rhizosphere soil around Bt maize HGK60 and the near-isogenic non-Bt maize ZD958 at four growth stages via a high-throughput sequencing technique. The results showed that HGK60 maize unleashed temporary effects on the bacterial and fungal diversity and richness during the study plant’s development, which would be restored after one cycle of plant cultivation due to the application of the same agricultural management. The differences of bacterial and fungal communities were marked by seasonality, while the different growth stage was the important factor as opposed to the cultivar contributing to the shifts in the bacterial and fungal communities’ structure. This study will provide useful information regarding the impact of Bt transgenic maize on the soil microbiome and a theoretical basis for the development of a safety assessment approach for Bt maize in China.

## 1. Introduction

Since insect-resistant and herbicide-tolerant biotech crops were developed and commercialized in 1996, the area of biotech crops worldwide has increased from 1.7 million hectares in 1996 to 190.4 million hectares in 2019, an increase of about 112 times [1]. Based on the great potential of biotech crops towards alleviating resource shortages, ensuring food security, and protecting the ecological environment, it is urgent to accelerate the cultivation and planting of a number of high-yield and high-quality biotech crop varieties with strong resistance to insects and tolerance to herbicides to cope with the complicated and changeable environment in China. Alongside the rapid development of biotech crops, the potential risks of biotech crops due to the diversification of exogenous gene sources and the unintended impacts on the environment should attract more attention [2,3,4].

Previous studies have shown that plants gather specific microbial communities and form unique soil microbial community structures [5]. Due to the interposition of an exogenous gene, biotech crops may affect the soil microbial community through their exudates and residues [6]. The rhizosphere is a narrow dynamic zone, in which complex interactions occur between root exudates and the soil microbiome [7]. Compared with that in bulk soil, microbial communities in rhizosphere soil are more sensitive to the unpredictability of biological development and the metabolism of biotech crops, and the bacterial and fungal community structure in the rhizosphere is often used as an early and sensitive indicator for assessing the effects of biotech crops on soil ecology [8,9].

Most early studies of soil microorganisms were based on a plate colony-counting technique, physiological approaches, and uncultivable molecular assays (including denaturing gradient gel electrophoresis, terminal restriction fragment-length polymorphisms, and pyrosequencing) [10,11,12,13]. However, these approaches have limitations; for instance, a maximum of approximately 5% of microbes can be cultured and isolated by traditional culture techniques [14]. Compared with the traditional methods, high-throughput sequencing has the characteristics of no culturing, high sensitivity, and a low detection limit, and has offered substantial insights into the presence of known and ‘unclassified’ (or ‘no rank’) microbes, which will be widely used in describing the relative abundance of microbial components and the structural variation in communities in rhizosphere soil.

Many efforts have been taken to assess the biosafety of biotech crops on soil microbial communities, but the results are still controversial. Bt maize and rice, for example, which were engineered to produce insecticidal proteins occurring naturally in *Bacillus thuringiensis* (Bt), significantly altered the fungal community structure and the infection efficiency of arbuscular mycorrhizal fungi (AMF) [15,16,17]. However, Bt cotton might stimulate soil bacterial and fungal growth and increase the diversity of soil microbial communities [18,19,20]. Non-Bt transgenic crops, including glyphosate-tolerant maize CC-2, high-methionine transgenic soybean ZD91, and transgenic BADH maize, had no significant effects on the structure and diversity of bacterial or fungal communities [9,21,22], while the drought-tolerant transgenic sugarcane did play a key role in strengthening plant–fungi interactions and enhancing beneficial fungal functions in the root-related area (rhizoplane and rhizosphere) [23,24]. Generally speaking, the ecological effects exerted by biotech crops on inhabitant soil bacteria or fungi are far from conclusive, which may be positive, neutral, or detrimental. It is essential to investigate the potential environmental risk of transgenic plants before their commercial release and the impacts of biotech crops on soil microbial communities need to be analyzed case-by-case.

In the previous study, we introduced the *cry1Ah* gene into maize by an *Agrobacterium*-mediated transformation and screened and obtained transgenic maize HGK60 with a high insect resistance. In the present study, we examined the alteration in the diversity and composition of bacterial and fungal communities in the rhizosphere around HGK60 and the near-isogenic non-Bt maize ZD958 at four growth stages and evaluated the ecological risk of HGK60 on microbial communities’ composition and diversity. The study will provide useful information associated with the impact of Bt transgenic maize on the soil microbiome and a theoretical basis for the development of a safety assessment approach for Bt maize in China.

## 2. Results

### 2.1. Illumina NovaSeq Sequencing Analysis of Rhizosphere Bacterial and Fungal Communities

**Figure 1:**
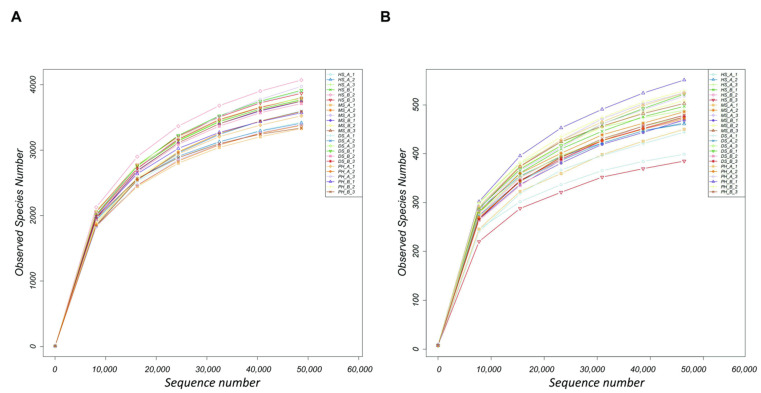
Rarefaction analysis. The rarefaction curve of the operational taxonomic units (OTUs) obtained from 16S rDNA (**A**) and ITS (**B**) amplicon sequencing in the rhizosphere of HGK60 and ZD958. The curves were named in the following form: “growth stage_cultivar_replicate”. HS—heading stage, MS—milk stage, DS—dough stage, PH—post-harvest stage, A—HGK60, and B—ZD958.

The V3V4 region of the 16S rRNA and ITS of 18S rRNA and 5.8S rRNA were amplified and sequenced on an Illumina NovaSeq PE250 platform to determine bacterial and fungal communities, respectively. In total, 1,488,810 and 1,774,766 high-quality sequences were obtained, with an average of 62,033 sequences per sample for 16S rDNA amplicon sequencing and 73,948 sequences per sample for ITS amplicon sequencing (Tables S1 and S2). The rarefaction curves showed no differences between HGK60 and ZD958, and the data were sufficient for revealing differences in the bacterial and fungal communities, if any, between the cultivars (Figure 1).

### 2.2. Richness and Diversity of Rhizosphere Bacteria

**Figure 2:**
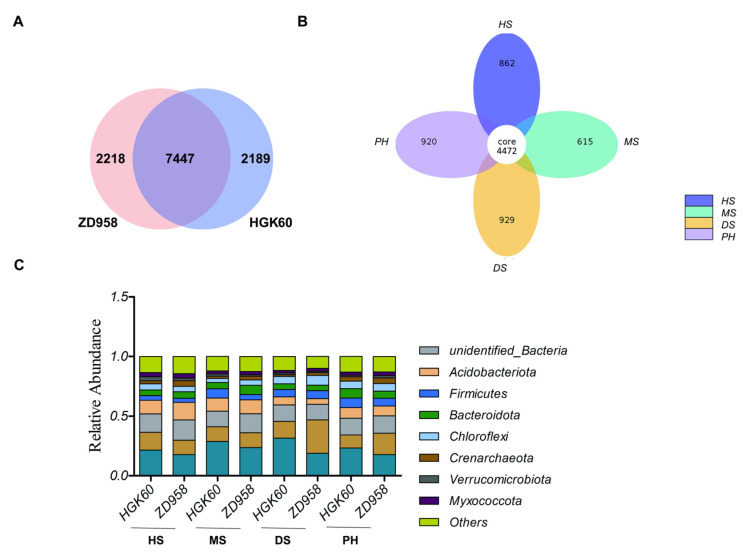
The composition of bacterial community in the rhizosphere soil of HGK60 and ZD958. (**A**) Venn diagram showing variable overlaps between HGK60 and ZD958; (**B**) Venn diagram showing variable overlaps between four growth stages; (**C**) relative read abundance of bacterial phyla within the communities. The numbers in (**A**) and (**B**) within the circles represent the operational taxonomic units (OTUs) numbers. HS—heading stage, MS—milk stage, DS—dough stage, and PH—post-harvest stage.

The 1,488,810 effective tags of all the samples were grouped into 11,854 operational taxonomic units (OTUs) at 97% similarity without a singleton. The analysis of the 11,854 OTUs showed that 9636 OTUs were observed in HGK60 and 9665 OTUs were observed in ZD958, with 7447 OTUs (62.8%) shared between them (Figure 2A). A total of 4472 OTUs (37.7%) exited in all the growth stages, while 862 OTUs were only observed at the heading stage, 615 OTUs at the milk stage, 929 OTUs at the dough stage, and 920 OTUs at post-harvest stage, which indicated that the number of OTUs existing in various developmental stages varied with the degree of maize development (Figure 2B).

Statistically significant differences in bacterial richness and diversity were observed for the richness estimators (OTUs, Chao1, and ACE) and diversity indices (Simpson, Shannon, and PD_whole_tree) at different growth stages (Table 1). There were also no significant differences in the rhizosphere bacterial richness estimators and diversity indices at almost all growth periods except that the richness estimator ACE in the rhizosphere soil of HGK60 was lower than that of ZD958, which indicated that there were no significant differences in the genetic relationship of rhizosphere bacteria species between HGK60 and ZD958.

### 2.3. Bacterial Community Composition

The phylogenetic classification based on 16S rRNA V3V4 hypervariable sequences at the phylum level for all the samples is shown in Figure S1. Among them, Proteobacteria was the dominant phylum with an average 22.89% relative abundance, followed by Actinobacteria (15.58%), Acidobacteria (9.66%), Bacteroidetes (5.88%), Firmicutes (5.85%), Chloroflexi (5.54%), Crenarchaeota (3.03%), Myxococcota (2.71%), and Verrucomicrobiota (1.79%). These phyla accounted for >70% of the relative bacterial abundance in total. Moreover, 14.67% of the relative abundance measure belonged to unclassified phyla (Figure 2C and Table S3). Further analysis showed that there were no significant differences in the relative abundance of the nine rhizosphere bacteria phyla between HGK60 and ZD958 during the most growth periods, except that the relative abundance values of Acidobacteriota were different between HGK60 and ZD958 at the heading stage, and the relative abundance values of Chloroflexi were different between HGK60 and ZD958 at the milk stage (Table S3). It seemed that the community compositions of a few bacteria at the phylum level were different between HGK60 and ZD958 at the early stages, but after a long period of planting, there were no significant differences, especially at the dough stage and post-harvest stage, which was consistent with the richness and diversity analysis of the rhizosphere bacteria.

**Figure 3:**
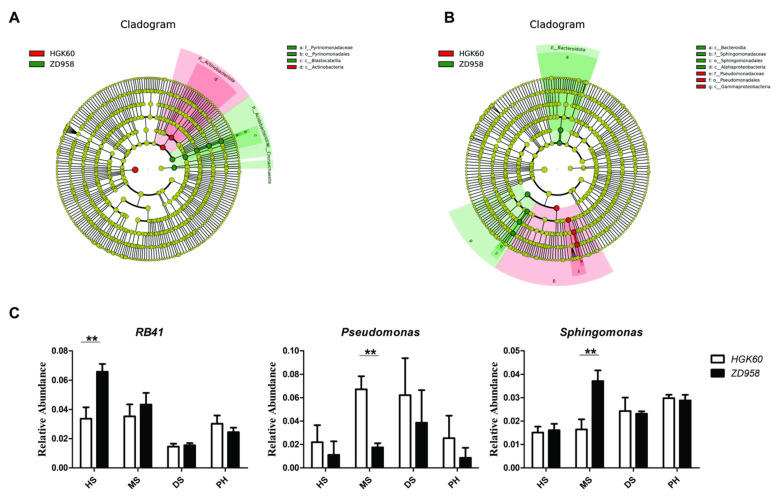
The identification of bacterial biomarkers resulting in the difference in composition of rhizosphere bacteria between HGK60 and ZD958 at early stages. (**A**,**B**) The bacterial biomarkers of HGK60 and ZD958 at the heading stage and milk stage; (**C**) the relative abundance of bacterial biomarkers. HS—heading stage, MS—milk stage, DS—dough stage, and PH—post-harvest stage. ** *p* value < 0.01.

In order to further identify the bacterial biomarkers at the genus level causing the difference in the composition of the rhizosphere bacteria between HGK60 and ZD958 at the early stages, the linear discriminant analysis effect size (LEFSE, Wilcoxon rank sum test, *p* < 0.05, and LDA score > 4) was determined at the heading stage and milk stage (Figure 3). At the heading stage, the *RB41* genus, which was classified into the Acidobacteriota phylum, Blastocatellia class, Pyrinomonadales order, and Pyrinomonadaceae family, was the bacterial biomarker of ZD958, and the relative abundance of *RB41* in the rhizosphere soil of ZD958 was higher than that of HGK60 (Figure 3A). At the milk stage, *Pseudomonas* belonging to the Proteobacteria phylum, Gammaproteobacteria class, Pseudomonadales order, and Pseudomonadaceae family was the bacterial biomarker in the rhizosphere soil of HGK60, while *Sphingomonas* belonging to the Proteobacteria phylum, Alphaproteobacteria class, Sphingomonadales order, and Sphingomonadaceae family comprised the biomarkers in the rhizosphere soil of ZD958 (Figure 3B). To further clarify the difference in the bacterial community compositions between HGK60 and ZD958, the relative abundances of all the biomarkers detected above were analyzed, especially at the dough stage and the post-harvest stage (Figure 3C). The results showed that all the bacterial biomarkers had no significant differences between HGK60 and ZD958 at the dough stage and post-harvest stage. This suggested that HGK60 had no effect on the bacterial communities’ composition after a long-term planting and that the difference of a few bacteria during planting was irregular, which might have been caused by accidental factors.

To gain a further insight into the impact of cultivars on the bacterial community at the genus level, the relative percentages of key genera for all the samples were depicted in heat maps (Figure S2). Thirty-five major genera were identified and there were significant differences in the relative abundances of some genera, which only appeared in a certain growth period. Compared with ZD958, the relative abundance of *Pseudomonas*, *Streptococcus*, *Enhydrobacter,* and *Vibrio* in the rhizosphere of HGK60 were lower and the relative abundance of *Sphingomonas* was higher at the milk stage; the relative abundances of *RB41*, *Bacillus,* and *Nocardioides* were lower in the rhizosphere of HGK60 at the heading stage but higher at the dough stage. Moreover, we found that the presence of the mange bacteria genera was significantly different among the three replicates. For example, the relative abundances of *Lactobacillus* and *Acetobacter* were higher in the replicate HGK60A52 than those in HGK60A51 and HGK60A53, and the relative abundances of *Bacteroides* and *Prevotella* were higher in the replicate ZD958B42 than those in ZD958B41 and ZD958B43. All the results indicated that many variations occur by chance among individuals and that bacterial composition is variable and affected by multiple factors. The cluster analysis manifested that the core genera of bacterial communities were similar between HGK60 and ZD958, although there were some differences in different samples.

### 2.4. Richness and Diversity of Rhizosphere Fungus

**Figure 4:**
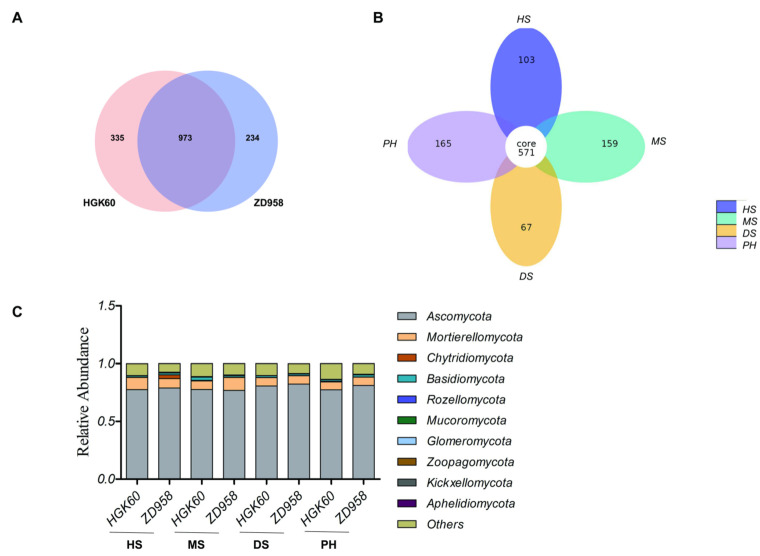
The composition of fungal community in the rhizosphere soil of HGK60 and ZD958. (**A**) Venn diagram showing variable overlaps between HGK60 and ZD958; (**B**) Venn diagram showing variable overlaps between four growth stages; (**C**) relative read abundance of fungal phyla classes within the communities. The numbers in (**A**,**B**) within the circles represent the operational taxonomic units (OTUs). HS—heading stage, MS—milk stage, DS—dough stage, and PH—post-harvest stage.

In order to study the fungal richness and diversity of each sample, the 1,774,766 effective tags of all the samples were grouped into 1542 OTUs at 97% similarity without singletons. An analysis of 1542 OTUs showed that 1308 OTUs were observed in HGK60 and 1207 OTUs were observed in ZD958, which shared 973 OTUs (Figure 4A). A total of 571 OTUs (37.03%) exited in all the growth stages, and 103 OTUs exited at the heading stage, 159 OTUs at the milk stage, 67 OTUs at the dough stage, and 165 OTUs at the post-harvest stage, which indicated that the number of OTUs varied with different developmental stages (Figure 4B).

Statistically significant differences in richness and diversity were observed for the OTUs, Chao1, ACE, Simpson, Shannon, and PD_whole_tree at different growth stages (Table 1). The rhizosphere fungal richness estimators (OTUs, Chao1, and ACE) and diversity indices (Shannon, Simpson, and PD_whole_tree) were similar between HGK60 and ZD958 in all growth periods.

### 2.5. Fungal Community Composition

The phylogenetic classification based on the ITS amplification sequences at the phylum level for all samples is shown in Figure S3. Among them, Ascomycota was the dominant phylum with an average relative abundance of 79.08%, followed by Mortierellomycota (8.20%), Basidiomycota (1.66%), and Chytridiomycota (0.65%) (Figure 4C and Table S4). Further analysis showed that there were no significant differences in the relative abundance of the four main rhizosphere fungal phyla between HGK60 and ZD958 during all growth periods (Table S5). Based on the fact that Ascomycota was the prevalent phylum accounting for >75% of the relative fungal abundance in total, the top 10 classes belonging to Ascomycota, including Sordariomycetes, Dothideomycetes, Eurotiomycetes and Leotiomycetes, were analyzed (Table S6). There were no significant differences in the relative abundance of the four rhizosphere fungal classes between HGK60 and ZD958 during all growth periods and the relative abundance varied similarly in the rhizosphere of both HGK60 and ZD958 with different growth stages. All the results indicated that the fungal community composition at the phylum level showed no significant difference between HGK60 and ZD958.

To gain an insight into the impact of cultivars on the fungal communities at the genus level, the relative percentages of key genera for all samples were shown in heat maps (Figure S4). There were 35 major genera, and 24 genera belonged to Ascomycota, which was consistent with the fact that Ascomycota was the dominant phylum. *Talaromyces*, *Fusarium*, *Cladosporium*, *Mortierella*, *Chaetomium,* and *Bipolaris* were the core fungal genera with high relative abundance in the rhizosphere soil of both HGK60 and ZD958. Compared with ZD958, the relative abundance of *Bipolaris* was lower at the dough stage and the relative abundances of *Fusarium* and *Chaetomium* were higher at the dough stage and milk stage, respectively. The cluster analysis manifested that the core genera of the fungal communities were similar between HGK60 and ZD958, although there were some differences in different samples.

The Arbuscular mycorrhizal fungi (AMF) constitute a group of non-target microorganisms that can form mutualistic symbioses with the roots of most plant species, provide nutrients, and enhance the disease resistance and stress tolerance of plants [25]. AMF are more sensitive to changes in the physiology of the host plant than other soil microorganisms and can be employed as a potential key non-target microorganism to be monitored in the assessment of the environmental influence of transgenic plants [26]. Thus, we focused on the effect of cultivars on AMF. Fifteen genera belonging to Glomeromycete were detected in the rhizosphere soil, and *Glomus* was the dominant genus of AMF. Further analysis showed that there were no significant differences in the relative abundances of all the AMF genera between HGK60 and ZD958 (Table S7). HGK60 had no influence on the communities of AMF during all the growth periods.

### 2.6. Effects of Cultivars and Growth Stages on the Rhizosphere Bacterial and Fungal Communities

**Figure 5:**
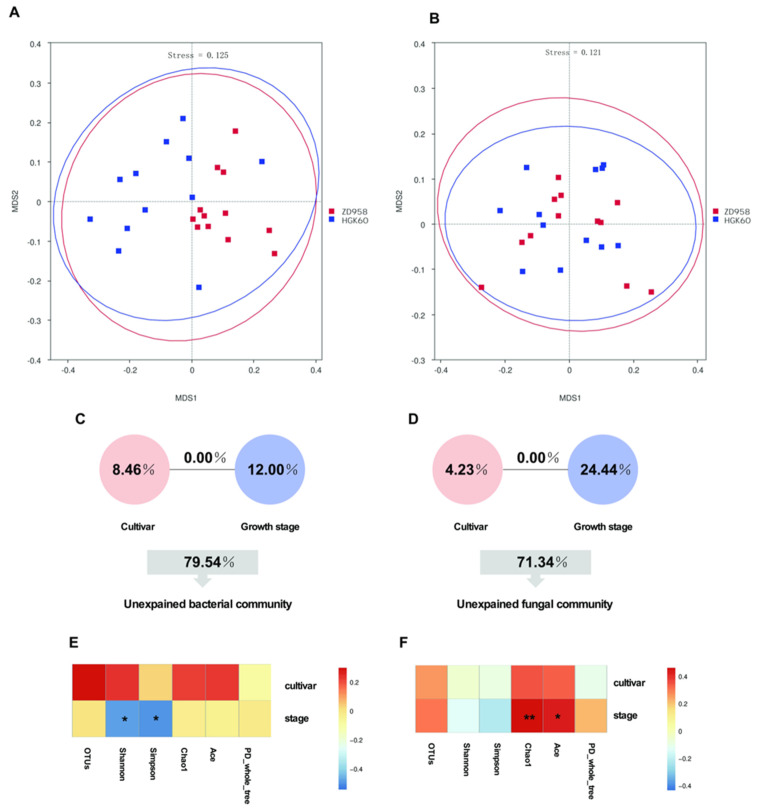
Effects of cultivar and stage on the rhizosphere bacterial and fungal communities. (**A**,**B**) Non-metric multidimensional scaling (NMDS) of bacterial and fungal community structures between cultivars, respectively; (**C**,**D**) variance-partitioning analysis (VPA) of the effects of cultivar, growth stage, and the interactions among these factors on the bacterial and fungal community structure. Circles show the percentage of variation caused by each factor alone. The unexplained variation is depicted in the squares at the bottom of the figure; (**E**,**F**) variance-partitioning analysis (VPA) of the effects of cultivar, growth stage, and the interactions among these factors on the bacterial and fungal community structures, respectively. ** *p* value < 0.01. * *p* value < 0.05.

Non-metric multidimensional scaling (NMDS) was performed to show the similarity of the bacterial and fungal community compositions and compare the bacterial and fungal community differences between cultivars (Figure 5). The NMDS ordination of the bacterial and fungal composition showed acceptable stress levels of 0.125 and 0.121, respectively, which indicated a good representation of the bacterial and fungal taxonomic compositions. Using cultivars as an explanatory variable, we found no significant differences in the rhizosphere bacterial and fungal communities between HGK60 and ZD958 (Figure 5A,B). To identify the effects of the cultivars and growth stages on the rhizosphere bacterial and fungal communities, a PERMANOVA analysis was performed (Table 2). According to the ADONIS value R^2^, the effect of the growth stages on the bacterial and fungal community compositions is greater than that of the cultivars, which was possibly caused by the degree of root exudation changing with plant maturity [27].

To quantify the relative contributions of the cultivars and growth stages to the total bacterial and fungal community based on OTU composition, the variance-partitioning analysis (VPA) and Spearman correlation of the alpha diversity and cultivars or growth stages were performed and determined. Variations in the bacterial or fungal community structures were partitioned among cultivars and growth stages, as well as the interactions among these variables. These variables explained 28.67% of the observed variations in bacterial composition and 20.46% of the observed variations in fungal composition, leaving 71.34% and 79.54% of the variations unexplained, respectively (Figure 5C,D). The cultivars explained 8.46% and 4.23% of the variations in bacterial and fungal composition, and growth stages accounted for 12.00% and 24.44%, respectively. The interactions between growth stages and cultivars accounted for 0% of the variations both in the bacterial and fungal compositions. Thus, the growth stage is the most important factor contributing to the shifts in the bacterial and fungal community structures. The bacterial diversity indices Shannon and Simpson were significantly correlated with the growth stages (Figure 5E and Table S8), and the fungal richness estimators, the Chao1 and ACE indices, were significantly correlated with the growth stages (Figure 5F and Table S9), which suggested that the effect of the growth stages on bacterial and fungal richness and diversity was greater than that of the cultivars.

**Figure 6:**
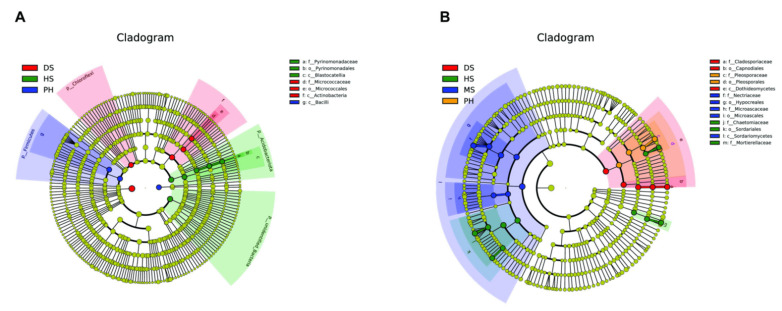
Linear discriminant analysis effect size (LEFSE) was employed based on the 10 compartments of data to identify the bacterial (**A**) and fungal (**B**) community biomarkers.

To further identify the bacterial and fungal community biomarkers of the growth stages, the linear discriminant analysis effect size (LEFSE, Wilcoxon rank sum test, *p* < 0.05, LDA score > 4) was employed based on the 10 compartments of data (Figure 6). Bacteria (Pyrinomonadaceae) and fungi (Chaetomiaceae) were enriched at the heading stage, fungi (Nectriaceae and Microascaceae) were enriched at the milk stage, bacteria (Micrococcaceae) and fungi (Cladosporiaceae) were enriched at the dough stage, and bacteria (Bacilli) and fungi (Pleosporaceae) were enriched at the post-harvest stage. All the results suggested that the difference of the bacterial and fungal communities was marked by seasonality, which is an environmental factor.

## 3. Discussion

To analyze the composition of the rhizosphere bacterial and fungal communities, the V3V4 region of 16S rRNA and ITS of 18S rRNA and 5.8S rRNA amplification sequencing were conducted on an Illumina NovaSeq PE250 platform. Lundin et al. found that 1000 denoised sequences per sample explained up to 90% of the trends in β-diversity [28]. Similarly, 5000 denoised sequences were sufficient to describe the trends in α-diversity. Since in our study, an average of 62,033 and 73,948 effective sequences per sample were used to analyze the bacterial and fungal communities, respectively, it is evident that our data sufficiently describe the patterns in the diversity and richness of the bacteria and fungi. Moreover, the lengths of the effective sequences in this study were 416 bp and 236 bp for bacteria and fungi, respectively, which were determined using the Illumina NovaSeq PE250 platform, while the number of sequence tags for each replicate ranged from 16,002 to 31,902 with an average length of 418 bp for the bacterial communities and 236 bp for the fungal communities, determined via the Miseq platform [29,30], which suggests that the NovaSeq PE250 platform achieves the same reading length as the Miseq platform, and greatly improves the throughput and sequencing quality. Furthermore, the number of fungal effective sequences per sample was greater than that of bacteria, while the number of OTUs was significant lower, which was consistent with the previous study [29]. This was because Ascomycota was the most dominant phylum of fungi with 79.08% relative abundance, while Proteobacteria was the dominant phylum of bacteria with only 22.89% relative abundance, which suggests more effective tags were clustered in Ascomycota.

Continuous monitoring throughout the growth period is indispensable to verifying whether transgenic plants have a persistent effect on the microbial community in the rhizosphere [30]. Among the soil organisms, bacteria and fungi are particularly important, as they are by far the most numerous and because of their essential roles in the functioning of soil [31]. In our study, the bacterial and fungal communities in the rhizosphere soil of HGK60 and ZD958 at four different growth stages were analyzed. The results showed that the core phyla and genera of HGK60 were similar to those of ZD958. Ascomycota and Proteobacteria were the most dominant phyla of fungi and bacteria in the rhizosphere soil of both HGK60 and ZD958, which was consistent with the previous results [17,21,29,30]. The members of Ascomycota, containing Sordariomycetes and Eurotiomycetes, could tolerate stressful conditions such as low nutrient availability, enabling them to achieve more efficient resource use in challenging environments [32]. Proteobacteria were related to the mediation of nitrogen transformation in root-associated soil [33] and the rapid growth rate of Proteobacteria was hypothesized to be the primary reason for this dominance [34]. Arbuscular mycorrhizal fungi (AMF) acquired carbohydrates from plants and provided phosphorus, nitrogen, and other mineral nutrients to plants via an extra radical mycelium network, especially when the presence of phosphorus was limited in the soil [35]. In addition, AMF reduced the harm to roots caused by the invasion of soil-borne plant pathogens and improved the capability of the host plant to balance water during heavy rain or drought conditions [36,37,38,39]. AMF were more sensitive to changes in the physiology of the host plant than other soil microorganisms and can be employed as a potential key non-target microorganism to be monitored in the assessment of the environmental influence of transgenic plants [26]. Fifteen genera belonging to Glomeromycete were detected in the rhizosphere soil, and *Glomus* was the dominant genus of AMF. There were no significant differences in the relative abundances of all the AMF genera between HGK60 and ZD958, which was in accordance with the results in which two Bt maize varieties, Bt11 and MO10, had no influence on the community of AMF [16].

Furthermore, we found that the difference of a few bacterial communities only existed during early development, which disappeared at the dough stage and post-harvest stage. Agricultural management was one of the major factors that determined community composition and abundance within the rhizosphere [40]. Biotech crop HGK60 unleashed temporary effects on bacterial diversity and richness during early plant development, which would be restored after one cycle of plant cultivation due to the application of the same agricultural management. Further analysis showed that the differences in the bacterial and fungal communities were marked by seasonality, and the different growth stage was the important factor instead of the cultivar contributing to the shifts in the bacterial and fungal communities’ structure. The results regarding the AMF further strengthen the conclusion that Bt maize HGK60 had no influence on the microbial community.

It has been reported that fungi can endure environmental changes that are less well-tolerated by other organisms, indicating a more stable structure of the fungal species than that of other microorganisms [41]. In our study, the bacterial richness estimators between HGK60 and ZD958 were different at a certain stage, while the fungal richness estimators of HGK60 were similar to those of ZD958 during all growth periods. This result suggested that the structure of the fungal species around the plant roots was more stable than that of bacteria in the rhizosphere soil, which was in accordance with the previous results [23,42].

Previous studies showed conflicting results regarding the influence of biotech crops on the composition of bacterial and fungal communities. Fungal diversity was affected in lettuce and *Arabidopsis thaliana* due to the plant genotype [24,43], while no significant differences with respect to fungal diversity between the non-biotech and biotech lines were found in potatoes, rice, and sugarcane [12,44,45]. Compared with the plant growth stage and year, the biotech trait was the least explanatory factor regarding variation in the bacterial composition [9,21,22], while BADH transgenic maize BZ-136 had a slight influence on bacterial diversity at different stages in neutral soil [30]. The conflicting results arise from different functional genes and the crop types. The microbial components in the soil are complex and fluctuate from time to time. An important issue to address is how to assess the effects of biotech crops on microbial components and whether the differences between transgenic and non-transgenic varieties are within the range of natural variation or not. Based on our data, it can be concluded that (1) there may to be need more non-transgenic cultivars, which can be used to judge whether the major differences that occur among non-transgenic cultivars are greater than those between non-transgenic and transgenic lines. (2) Continuous monitoring throughout a given growth period is an indispensable process for verifying whether transgenic plants have a persistent effect on the microbial communities in the rhizosphere. Season-related natural variation may be more significant than that of genetic manipulation. (3) Soil factors and agricultural management are the major factors that determine community composition and abundance within the rhizosphere [40]. Therefore, it is necessary to identify whether the microbial components of different soil plots are similar before planting and to apply the same agricultural management scheme during all periods.

So far, the evaluation of the impacts of biotech crops on their soil microenvironments have mostly focused on simple insect resistance, herbicide resistance, or other functional characteristics, while there have been few assessments related to stacked characteristics. Herbicide resistance genes such as *bar*, *EPSPS,* and *GAT* have been globally used in basic plant research and genetically engineered crops [46,47]. We have obtained a few stacked biotech crops with simultaneous resistance to herbicide and insects through co-transformation and conventional breeding. Therefore, we will conduct a safety assessment of stacked biotech maize with respect to the microbial components within the rhizosphere and clarify the impact of stacked-traits and herbicide usage on soil microbial communities, which will provide a theoretical basis for the development of a safety assessment approach for stacked-trait transgenic crops in China.

## 4. Materials and Methods

### 4.1. Plant and Soil Materials

Two maize lines, namely, the transgenic maize HGK60 and the near-isogenic non-Bt maize ZD958, were planted in Langfang, Hebei, China (116.71° E, and 39.52° N), in the summer of 2021. Langfang has a typical temperate continental monsoon climate with an average annual precipitation of 555.3 mm and temperature of approximately 11.9 °C. The examined soils belong to fluvo-aquic soil, which contains organic carbon, total nitrogen, alkaline nitrogen, available potassium, slow-acting potassium, and available phosphorus at concentrations of 18.4 g/kg, 1.10 g/kg, 114 mg/kg, 194 mg/kg, 942 mg/kg, and 27.0 mg/kg, respectively. The pH of the soil is 8.40–8.60. The total area of this experimental field was 600 m^2^, which was divided into 6 plots of 100 m^2^ with 3 replicate plots for HGK60 and ZD958. Maize was cultivated in accordance with the regular agronomic practices in China. Samples were taken when the plants were at the heading stage (HS), milk stage (MS), dough stage (DS), and post-harvest stage (PH). The rhizosphere soil of five plants from the same plot was shaken off and combined as a sample to ensure representativeness of soil. Then, the soil samples were stored at −80 °C for DNA extraction.

### 4.2. Total DNA Extraction, Amplification, and Illumina NovaSeq Sequencing

Total microbial genomic DNA was extracted from the soil samples using the FastDNA SPIN Kit for Soil (Menlo Park, CA, USA) according to the manufacturer’s instructions. The concentration of the extracted DNA was determined using a NanoDrop 2000 spectrophotometer (Thermo Fisher Scientifc, Waltham, MA, USA) and diluted to 1 ng/μL with sterile water. PCR amplification was performed with the total microbial DNA as the template using a GeneAmp PCR-System^®^ 9700 (Applied Biosystems, Foster City, CA, USA). The 16S rDNA V3V4 region of bacteria and the internal transcribed spacer (ITS) rDNA gene of fungi were amplified using the paired primers 338F-806R and ITS1F-ITS2, respectively. The primers were listed in Table S10. PCR reactions were conducted in triplicate for each sample using Phusion^®^ High-Fidelity PCR Master Mix with GC Buffer (New England Biolabs, Inc., Ipswich, MA, USA). Amplicons were extracted from 2% agarose gels and purified using the AxyPrep DNA Gel Extraction Kit (Axygen Biosciences, Union City, CA, USA), and quantified using QuantiFluor™-ST (Promega, WI, USA) according to the manufacturer’s instructions. TruSeq^®^ DNA PCR-Free Sample Preparation Kit (Illumina, DE, USA) was used for library construction, which was qualified by QUBIT and Q-PCR and run on the Illumina NovaSeq sequencing platform from Novegene bioinformatics technology Co., Ltd. (Beijing, China).

### 4.3. Sequences Processing and Bioinformatics Analysis

Based on the overlapping relationship between paired-end reads, the paired reads were merged using FLASH [48] into a sequence with a minimum overlapping length of 10 bp. Raw sequences were truncated based on the Fred algorithm if quality dropped below 20 over a sliding window of 25 bp, and low-quality reads with less than 75% of their original length were trimmed [49]. Then, the final effective tags were obtained by comparison with the species annotation database to detect the potential chimeras and remove them [50,51]. The assembled tags were clustered in operational taxonomic units (OTUs) at 97% similarity level using the UPARSE algorithm in Usearch version 7.0.1090 [52]. OTU representative sequences were taxonomically classified using Ribosomal Database Project (RDP) Classifer v.2.2 with a minimal 50% confidence estimate, referencing the SSUrRNA database for bacteria and fungi by using the methods of Mothur and SILVA138 [53,54]. Based on the taxonomical classification and the Bray–Curtis distances of all samples, a series of bacteria and fungi community-related analyses was then performed. The diversity (Shannon, Simpson, and PD_whole_tree indices) and richness (OTUs, Chao1, and ACE indices) calculated by Qiime Software were determined for the samples. A Venn diagram was produced to show the unique and shared OTUs present in the bacterial and fungal communities in rhizosphere soils. Nonmetric multidimensional scaling (NMDS) was also conducted using vegan software to determine the differences and similarities among the bacterial and fungal communities in rhizosphere soils associated with growth stages or maize species. Furthermore, NMPANOVA (nonparametric multivariate analysis, also known as PERMANOVA or Adonis) and variance-partitioning analysis (VPA) were conducted using vegan software to further confirm significant associations between two or more groups, including the maize species and growth stages. Finally, LEfSe was determined to support the high-dimensional taxonomical comparisons using LEfSe software.

## 5. Conclusions

The data from our study revealed that the bacterial and fungal communities’ diversity and structure in the rhizosphere of transgenic maize HGK60 were similar to those of ZD958. Moreover, different growth stages were the driving factors affecting the bacterial and fungal communities, and season-related natural variation may be a more significant factor than that of genetic manipulation. Compared with the previously published data, the llumina NovaSeq PE250 method had a higher throughput and a higher quality read cover, which gave us a comprehensive insight into the microbial community in agroecosystems.

## Figures and Tables

**Table 1:** Diversity index of rhizosphere bacteria and fungi in different groups.

	Stage	Group	OTUs	Chao1	ACE	Shannon	Simpson	PD_Whole_Tree
Bacteria	Heading stage	HGK60	3536.33 ± 199.92 a	3915.77 ± 237.40 a	3990.06 ± 262.29 a	10.07 ± 0.08 a	0.997 ± 0.000 a	279.56 ± 15.01 a
		ZD958	3904.33 ± 154.07 a	4372.87 ± 253.06 a	4421.24 ± 215.71 a	9.94 ± 0.08 a	0.996 ± 0.000 a	276.69 ± 3.21 a
	Milk stage	HGK60	3567 ± 32.36 a	3749.77 ± 40.44 a	3987.63 ± 30.20 a	9.55 ± 0.12 a	0.993 ± 0.002 a	288.78 ± 8.96 a
		ZD958	3696.33 ± 92.96 a	4296.01 ± 87.63 a	4179.12 ± 92.09 b	9.77 ± 0.10 a	0.995 ± 0.001 a	271.47 ± 11.28 a
	Dough stage	HGK60	3765.67 ± 153.82 a	4233.33 ± 145.37 a	4294 ± 158.83 a	9.53 ± 0.26 a	0.991 ± 0.003 a	299.36 ± 12.08 a
		ZD958	3808 ± 101.53 a	4225.11 ± 156.55 a	4303.94 ± 161.51 a	9.53 ± 0.10 a	0.987 ± 0.002 a	283.70 ± 12.36 a
	Post-harvest stage	HGK60	3696.67 ± 243.22 a	4291 ± 474.18 a	4334.02 ± 516.9 a	9.56 ± 0.19 a	0.994 ± 0.001 a	290.69 ± 19.93 a
		ZD958	3567.67 ± 226.53 a	3901.7 ± 272.47 a	3929.24 ± 285.9 a	9.89 ± 0.10 a	0.995 ± 0.001 a	261.91 ± 6.07 a
Fungi	Heading stage	HGK60	449.67 ± 46.06 a	507.82 ± 41.99 a	509.96 ± 39.22 a	5.48 ± 0.24 a	0.952 ± 0.001 a	133.21 ± 20.27 a
		ZD958	450.33 ± 58.29 a	539.24 ± 94.82 a	533.78 ± 84.02 a	5.09 ± 0.3 a	0.931 ± 0.006 a	110.78 ± 12.82 a
	Milk stage	HGK60	487 ± 37.51 a	602.09 ± 41.89 a	603.37 ± 36.54 a	5.45 ± 0.09 a	0.944 ± 0.003 a	144.53 ± 24 a
		ZD958	493.67 ± 21.57 a	607.99 ± 16.28 a	606.46 ± 22.37 a	5.47 ± 0.07 a	0.953 ± 0.001 a	129.14 ± 3.91 a
	Dough stage	HGK60	460.67 ± 15.63 a	546.64 ± 34.67 a	555.84 ± 22.91 a	5.17 ± 0.17 a	0.931 ± 0.015 a	109.72 ± 13.51 a
		ZD958	491.67 ± 26.31 a	640 ± 82.26 a	634.44 ± 68.3 a	5.08 ± 0.06 a	0.949 ± 0.001 a	122.49 ± 6.22 a
	Post-harvest stage	HGK60	504.67 ± 28.45 a	651.4 ± 49.9 a	646.66 ± 56.59 a	5.32 ± 0.09 a	0.925 ± 0.007 a	148.79 ± 16.44 a
		ZD958	519.67 ± 36.9 a	648.81 ± 45.53 a	642.21 ± 48.03 a	5.26 ± 0.35 a	0.922 ± 0.036 a	126.48 ± 8.9 a

Note: OTUs: the number of operational taxonomic units; Chao1: the total number of species in the estimated community; Ace: the number of OTUs in the estimated community; Shannon and Simpson: the diversity and evenness of species distribution in the community; PD_whole_Tree: the genetic relationship of species in the community. All values were given as mean ± SD, and the same letters mean there were no significant difference between HGK60 and ZD958 at *p* < 0.05 level according to the *t*-test.

**Table 2:** PERMANOVA analysis results of the association of bacterial and fungal community structures.

		F. Model	R^2^	Pr (>F)
Bacteria	Cultivars	3.226	0.10331	0.001
	Growth stages	3.9504	0.28318	0.004
Fungi	Cultivars	1.4561	0.04943	0.149
	Growth stages	4.4012	0.30561	0.002

## Data Availability

All sequences have been deposited in the National Genomic Data Center (BioProject accession No. PRJCA010214 for 16S and PRJCA010301 for ITS).

## Supplementary Materials

The following supporting information can be downloaded at: https://www.mdpi.com/article/10.3390/plants11212824/s1, Figure S1: The phylogenetic classification based on 16S rRNA V3V4 hypervariable sequences at the phylum level for all samples; Figure S2: Heat maps of the soil rhizosphere bacterial composition at the genus level; Figure S3: The phylogenetic classification based on internal transcribed spacer (ITS) sequences at the phylum level for all samples; Figure S4: Heat maps of the soil rhizosphere fungal composition at the genus level; Table S1: Data statistics and quality control 16S rDNA amplicon sequencing; Table S2: Data statistics and quality control for ITS amplicon sequencing; Table S3: Comparative analysis of the relative abundance (%) of main bacteria phyla in the rhizosphere of HGK60 and ZD958; Table S4: The relative abundance of main fungi phyla in the rhizosphere soil of HGK60 and ZD958; Table S5: Comparative analysis of the relative abundance of top 4 fungal phyla between HGK60 and ZD958; Table S6: Comparative analysis of the relative abundance of classes belonged to Ascomycota between HGK60 and ZD958; Table S7: Comparative analysis of the relative abundance (×10^4^) of arbuscular mycorrhizal fungi (AMF) between HGK60 and ZD958; Table S8: The Spearman correlation of bacterial alpha diversity and cultivars or growth stages; Table S9: The Spearman correlation of fungal alpha diversity and cultivars or growth stages; Table S10: The primers for PCR amplification.
