# Supplementary material for: Effects of Insect-Resistant Maize HGK60 on Community Diversity of Bacteria and Fungi in Rhizosphere Soil
Source: Plants (Basel). 2022 Oct 24;11(21):2824. doi: 10.3390/plants11212824 (PMC9653938; doi:10.3390/plants11212824)
Supplement: Supplementary file 1 [file plants-11-02824-s001.zip › supplementary materials/Figure S1 The phylogenetic classification based on 16S rRNA V3V4 hypervariable sequences at the phylum level for all samples.pdf]

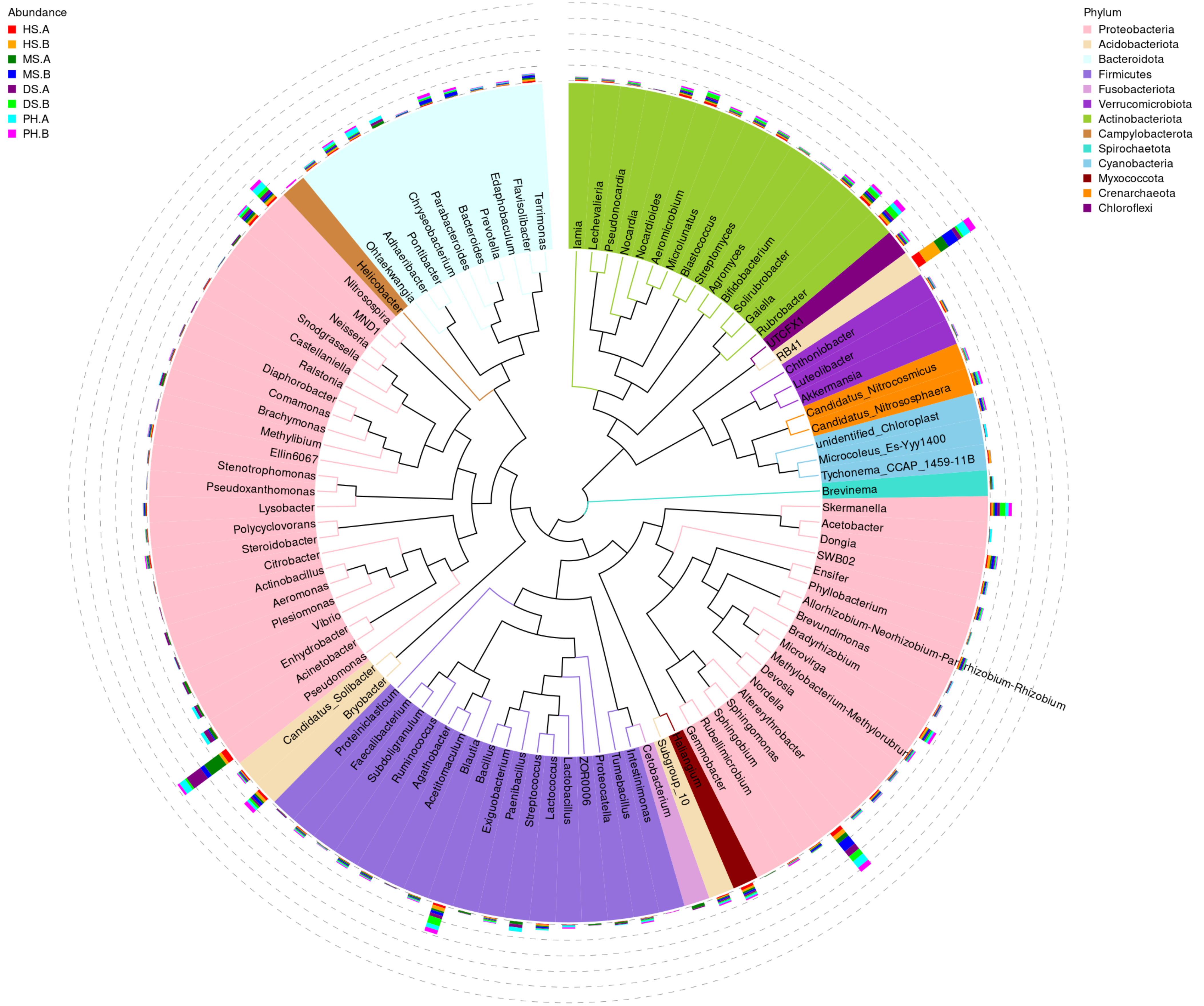

Abundance
HS.A
HS.B
MS.A
MS.B
DS.A
DS.B
PH.A
PH.B
Phylum
Proteobacteria
Acidobacteriota
Bacteroidota
Firmicutes
Fusobacteriota
Verrucomicrobiota
Actinobacteriota
Campylobacterota
Spirochaetota
Cyanobacteria
Myxococcota
Crenarchaeota
Chloroflexi
Iamia
Lechevalieria
Pseudonocardia
Nocardia
Nocardioides
Aeromicrobium
Microlunatus
Blastococcus
Streptomyces
Agromyces
Bifidobacterium
Solirubrobacter
Gaiella
Rubrobacter
JTCFX1
RB41
Chthoniobacter
Luteolibacter
Akkermansia
Candidatus_Nitrocosmicus
Candidatus_Nitrososphaera
unidentified_Chloroplast
Microcoleus_Es-Yyy1400
Tychonema_CCAP_1459-11B
Brevinema
Skermanella
Acetobacter
Dongia
SWB02
Ensifer
Phyllobacterium
Allorhizobium-Neorhizobium-Pararhizobium-Rhizobium
Brevundimonas
Bradyrhizobium
Microvirga
Methylobacterium-Methylorubrum
Devosia
Nordella
Altererythrobacter
Sphingomonas
Sphingobium
Rubellimicrobium
Gemmobacter
Haliangium
Subgroup_10
Cetobacterium
Intestinimonas
Tumebacillus
Proteocatella
ZOR0006
Lactobacillus
Lactococcus
Streptococcus
Paenibacillus
Exiguobacterium
Bacillus
Blautia
Aceitomaculum
Agathobacter
Ruminococcus
Subdoligranulum
Faecalibacterium
Proteiniclasticum
Bryobacter
Candidatus_Solibacter
Pseudomonas
Acinetobacter
Enhydrobacter
Vibrio
Plesiomonas
Aeromonas
Actinobacillus
Citrobacter
Steroidobacter
Polycyclovorans
Lysobacter
Pseudoxanthomonas
Stenotrophomonas
Ellin6067
Methylibium
Brachymonas
Comamonas
Diaphorobacter
Ralstonia
Castellaniella
Snodgrassella
Neisseria
MND1
Nitrosospira
Helicobacter
Ohtaekwangia
Adhaeribacter
Pontibacter
Chryseobacterium
Parabacteroides
Bacteroides
Prevotella
Edaphobaculum
Flavisolibacter
Terrimonas
