# Supplementary material for: Effects of Insect-Resistant Maize HGK60 on Community Diversity of Bacteria and Fungi in Rhizosphere Soil
Source: Plants (Basel). 2022 Oct 24;11(21):2824. doi: 10.3390/plants11212824 (PMC9653938; doi:10.3390/plants11212824)
Supplement: Supplementary file 1 [file plants-11-02824-s001.zip › supplementary materials/Figure S3 The phylogenetic classification based on internal transcribed spacer (ITS) sequences at the phylum level for all samples.pdf]

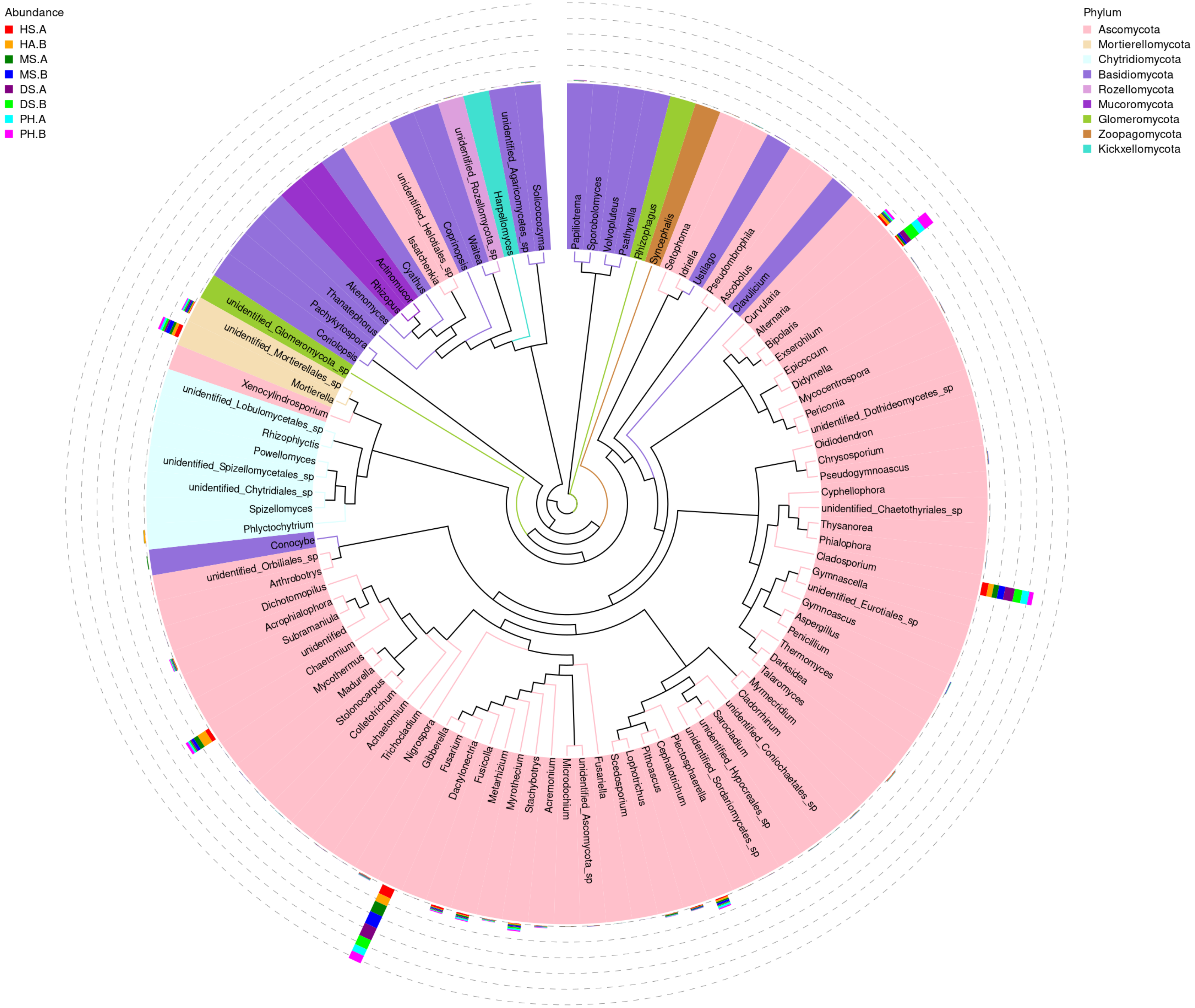

Abundance
HS.A
HA.B
MS.A
MS.B
DS.A
DS.B
PH.A
PH.B
Phylum
Ascomycota
Mortierellomycota
Chytridiomycota
Basidiomycota
Rozellomycota
Mucoromycota
Glomeromycota
Zoopagomycota
Kickxellomycota
Papiliotrema
Sporobolomyces
Volvopluteus
Psathyrella
Rhizophagus
Syncephalis
Setophoma
Idriella
Ustilago
Pseudombrophila
Ascobolus
Clavulicium
Curvularia
Alternaria
Bipolaris
Exserohilum
Epicoccum
Didymella
Mycocentrospora
Periconia
unidentified_Dothideomycetes_sp
Oidiodendron
Chrysosporium
Pseudogymnoascus
Cyphellophora
unidentified_Chaetothyriales_sp
Thysanorea
Phialophora
Cladosporium
Gymnascella
unidentified_Eurotiales_sp
Gymnoascus
Aspergillus
Penicillium
Thermomyces
Darksidea
Talaromyces
Myrmecridium
Cladorrhinum
unidentified_Coniochaetales_sp
Sarocladium
unidentified_Hypocreales_sp
unidentified_Sordariomycetes_sp
Plectosphaerella
Cephalotrichum
Pithoascus
Lophotrichus
Scedosporium
Fusariella
unidentified_Ascomycota_sp
Microdochium
Acremonium
Stachybotrys
Myrothecium
Metarhizium
Fusicolla
Dactylonectria
Fusarium
Gibberella
Nigrospora
Trichocladium
Achaetomium
Colletotrichum
Stolonocarpus
Madurella
Mycothermus
Chaetomium
unidentified
Subramaniula
Acrophialophora
Dichotomopilus
Arthrobotrys
unidentified_Orbiliales_sp
Conocybe
Phlyctochytrium
Spizellomyces
unidentified_Chytridiales_sp
unidentified_Spizellomycetales_sp
Powellomyces
Rhizophlyctis
unidentified_Lobulomycetales_sp
Xenocylindrosporium
Mortierella
unidentified_Mortierellales_sp
unidentified_Glomeromycota_sp
Coriolopsis
Pachykytospora
Thanatephorus
Akenomyces
Rhizopus
Actinomucor
Cyathus
Issatchenkia
unidentified_Helotiales_sp
Coprinopsis
Waitea
unidentified_Rozellomycota_sp
Harpellomyces
unidentified_Agaricomycetes_sp
Solicoccozyma
