# Supplementary figures and images for: Effects of Insect-Resistant Maize HGK60 on Community Diversity of Bacteria and Fungi in Rhizosphere Soil
Source: Plants (Basel). 2022 Oct 24;11(21):2824. doi: 10.3390/plants11212824 (PMC9653938; doi:10.3390/plants11212824)

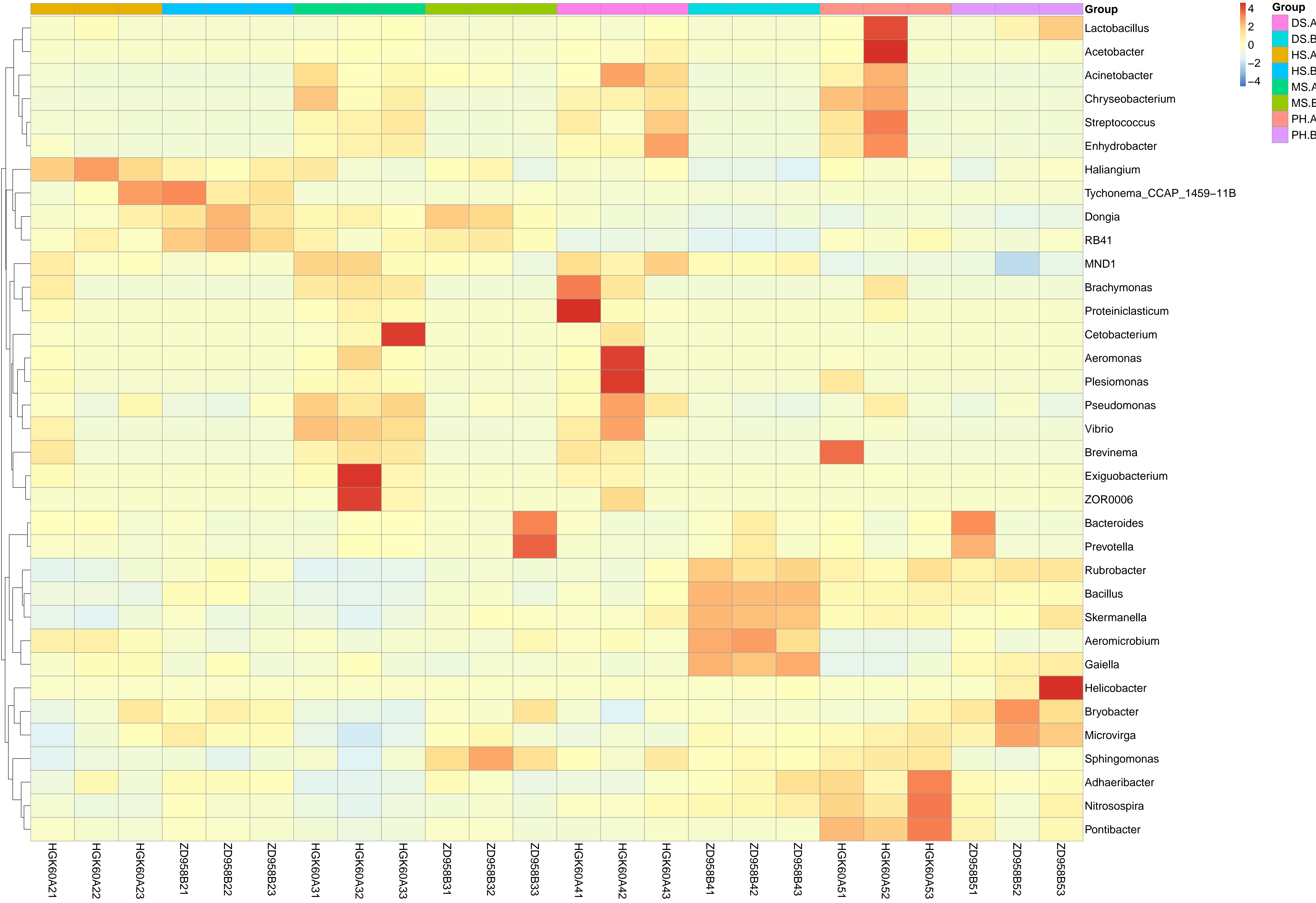

Supplement: Supplementary file 1 [file plants-11-02824-s001.zip › supplementary materials/Figure S2 Heat maps of the soil rhizosphere bacterial composition at the genus level.pdf]

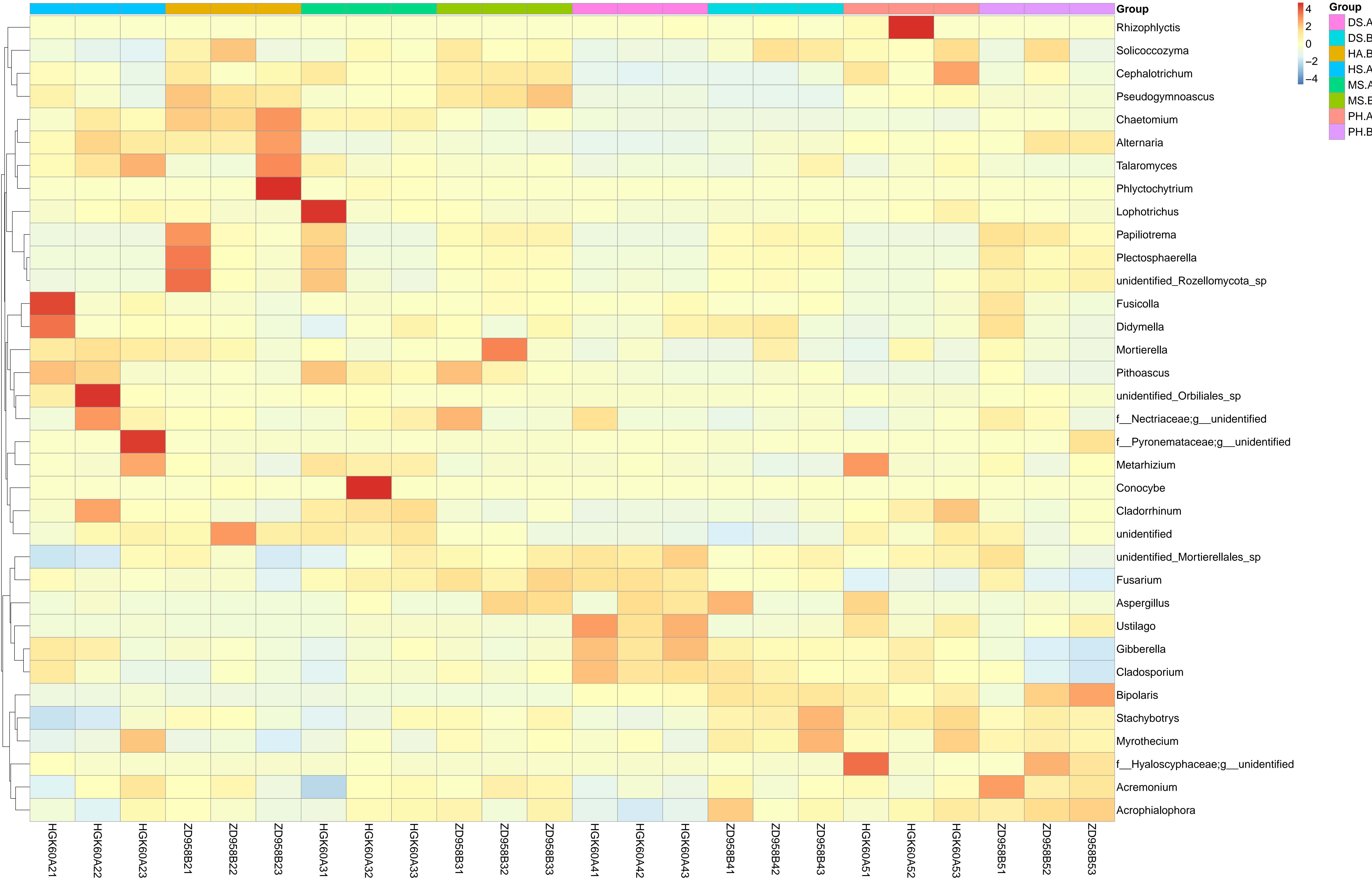

Supplement: Supplementary file 1 [file plants-11-02824-s001.zip › supplementary materials/Figure S4 Heat maps of the soil rhizosphere fungal composition at the genus level.pdf]
